# Temperature‐Related Changes in Avian Nestling Provisioning: A Global Analysis

**DOI:** 10.1111/gcb.70871

**Published:** 2026-04-17

**Authors:** Elke Molenaar, Kat Bebbington, Kevin D. Matson, Sjouke A. Kingma

**Affiliations:** ^1^ Behavioural Ecology Group, Department of Animal Sciences Wageningen University and Research Wageningen the Netherlands; ^2^ Wildlife Ecology and Conservation Group, Department of Environmental Sciences Wageningen University and Research Wageningen the Netherlands

**Keywords:** birds, climate change, life‐history traits, meta‐analysis, nestling provisioning, parental care, temperature

## Abstract

Temperature‐related changes in behaviour can negatively affect population persistence. One way in which species may be affected is in their ability to provide parental care: if parents are constrained, either physiologically or due to food limitation to provision nestlings, offspring development and survival may be impacted. Empirical studies have shown temperature‐related changes in nestling provisioning in some, but not other species, yet the reason for this difference among species remains unclear. To predict the consequences of global change, it is important to understand what makes a particular species vulnerable to temperature‐induced changes in parental care. Here, we used 47 effect sizes from published studies that quantified the relationship between ambient temperature and nestling provisioning rate in 39 bird species to determine how latitude, climate, breeding system, nestling diet and body mass explain variation in this relationship. Across all species, nestling provisioning rate was negatively correlated with temperature, regardless of latitude or breeding climate. Cooperative breeders provisioned less when temperatures rose, which contradicts prevailing predictions that helpers can mitigate adverse conditions but may also be because helpers can compensate when conditions become more suitable. This pattern was different in species with biparental care, which showed a wider range of responses. Moreover, provisioning was more impacted by temperature in larger‐bodied species and in species with offspring that are not exclusively insectivorous, presumably either because food is more difficult to collect or because the energetic costs of provisioning are higher when it is warmer. Unless reduced provisioning is offset by higher quality food, parents apparently do not systematically buffer the effects of increased temperature, leaving nestlings to carry the costs. Knowing which characteristics make birds vulnerable to increasing temperatures can help in shaping future studies of climate change ecology and predicting differential effects of climate change on avian populations on a global scale.

## Graphical abstract

Using a global meta‐analysis, we examined how ambient temperature influences nestling provisioning rates in birds. We synthesised effect sizes from published studies that accounted for key ecological covariates and tested whether species traits and breeding context explained differences in temperature sensitivity. We revealed that across all studies, increasing temperatures are associated with an overall reduction in nestling provisioning in birds. Species traits, rather than latitude or breeding climate, determined vulnerability, with larger‐bodied and non‐insectivorous species showing stronger responses.

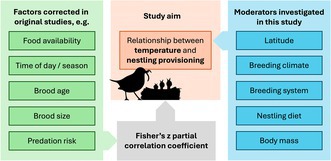

## 1 Introduction

Ongoing climate change necessitates the study of organismal responses to increased temperatures in order to understand and predict how populations, species and ecosystems will be affected. Warmer land areas carry new ecological opportunities and challenges for terrestrial life: some animals will benefit from increased temperatures while others will be pushed out of their evolved thermal optima (Şekercioğlu et al. 2012). For example, climate change has been linked to population declines of several bird species (Bellard et al. 2012; Northrup et al. 2019). However, the underlying mechanisms driving these declines remain poorly understood. Moreover, species differ markedly in their responses to climate change. For instance, some species show strong reductions in reproductive success or survival under high temperatures, whereas others appear more resilient (Halupka et al. 2023; Nomano et al. 2019). This variation makes it difficult to predict how climate change will influence key demographic processes and, ultimately, population dynamics. Identifying which factors drive variation in response to climate change is essential if we are to predict the effects of climate change and implement species‐specific interventions.

Reproductive success, a central element of population maintenance, can act as an early warning system for impacts of climate change. When confronted with high temperatures, many bird species exhibit reduced reproductive success (e.g., due to overheating or starvation) or skip reproduction entirely (Crick 2004; García‐Navas and José Sanz 2012; Loning et al. 2023). Parental care, a critical component for nestling development and key to reproductive success, can become particularly costly under challenging conditions (Nomano et al. 2019). Thus, increasing temperatures can affect both parents and offspring, but who bears the main costs depends on decisions and behaviours of the parents. Specifically, self‐maintenance behaviours of parents, including foraging, drinking, thermoregulation and heat avoidance directly trade off with the care they provide their offspring (Cunningham et al. 2021). Furthermore, under changing temperatures, food may become scarcer, or parents may need to increase their effort to obtain prey (Visser et al. 2012). Even short‐term changes in provisioning (quantity or quality) can lead to nutritional stress in nestlings, and this nutritional stress can have long‐term and transgenerational effects on physiology and behaviour (Monaghan 2008; Naguib and Gil 2005). Overall, the ability of birds to care for their offspring is thus strongly affected by temperature, yet there also is substantial variation among species in these impacts (Du Plessis et al. 2012; Trapote et al. 2023; Wiley and Ridley 2016). The overall patterns in the relationship between temperature and nestling provisioning rate are poorly understood (Lejeune et al. 2019; Vincze et al. 2017), and most likely relate to environmental, ecological and evolutionary (i.e., life‐history) differences.

Provisioning rates reflect a part of parental effort and are a widely used quantitative indicator of parental care in within‐ and among‐species comparisons. Provisioning rates show considerable variation across different social and ecological conditions (Vincze et al. 2017; Wiley and Ridley 2016). For example, nestling age and brood size show strong positive relationships with provisioning rates (Bowers et al. 2014; Browning et al. 2012; Wiebe and Slagsvold 2014). Furthermore, the frequency of provisioning depends on habitat quality and with that of food availability (Tremblay et al. 2005) and predation risk (Goullaud et al. 2018; Leniowski and Węgrzyn 2018). Different features of species, including how and where they live, could also potentially impact the relationship between temperature and provisioning. For example, birds at lower latitudes or those breeding in warmer climates may operate closer to their thermal maxima and therefore may be less able to maintain their provisioning rates if temperatures become even warmer (AlRashidi et al. 2010; Van De Ven et al. 2020). For birds breeding at higher latitudes or in colder climates, an increase in temperature may actually lower the energetic costs of care (Reid et al. 2000), allowing for higher provisioning rates. Moreover, a second mechanism, variation in pace of life, might underlie latitudinal effects on the relation between temperature and provisioning effort. At lower latitudes, bird species often exhibit a slower pace of life and invest more in self‐maintenance (Hille and Cooper 2015; McNamara et al. 2008; Wiersma et al. 2007). Such species might not be under strong selection to preserve their current nesting attempt if temperatures become suboptimal, instead favouring future efforts. Furthermore, the cost of offspring care per individual depends on the number of caregivers (Cram et al. 2015; Nomano et al. 2019). Thus, in breeding systems with multiple individuals providing parental care (cooperative breeders), load‐lightening through sharing any increase in effort may facilitate the upkeep of provisioning rates under difficult circumstances (Van Boheemen et al. 2019). Therefore, cooperatively breeding species may be better able to maintain or increase provisioning rates in response to challenging conditions, such as an increase in temperature. In addition, diet type may shape the response of nestling provisioning rate to increased temperature, since some types of food might be less abundant or more difficult or energy‐consuming to obtain when temperatures are higher (Arbeiter et al. 2016). Lastly, the relationship between nestling provisioning and temperature may depend on body mass. Larger birds have lower surface area to volume ratios than smaller birds, so they become more susceptible to overheating at high temperatures (McKechnie et al. 2021; Smit et al. 2016; Watson and Kerr 2025; Weathers 1981). Thus, body mass may also affect the ability to provision offspring when temperatures rise. While individual studies have suggested a role for these factors in determining how parents respond to temperature increases, comprehensive global analyses are still missing.

**FIGURE 1:**
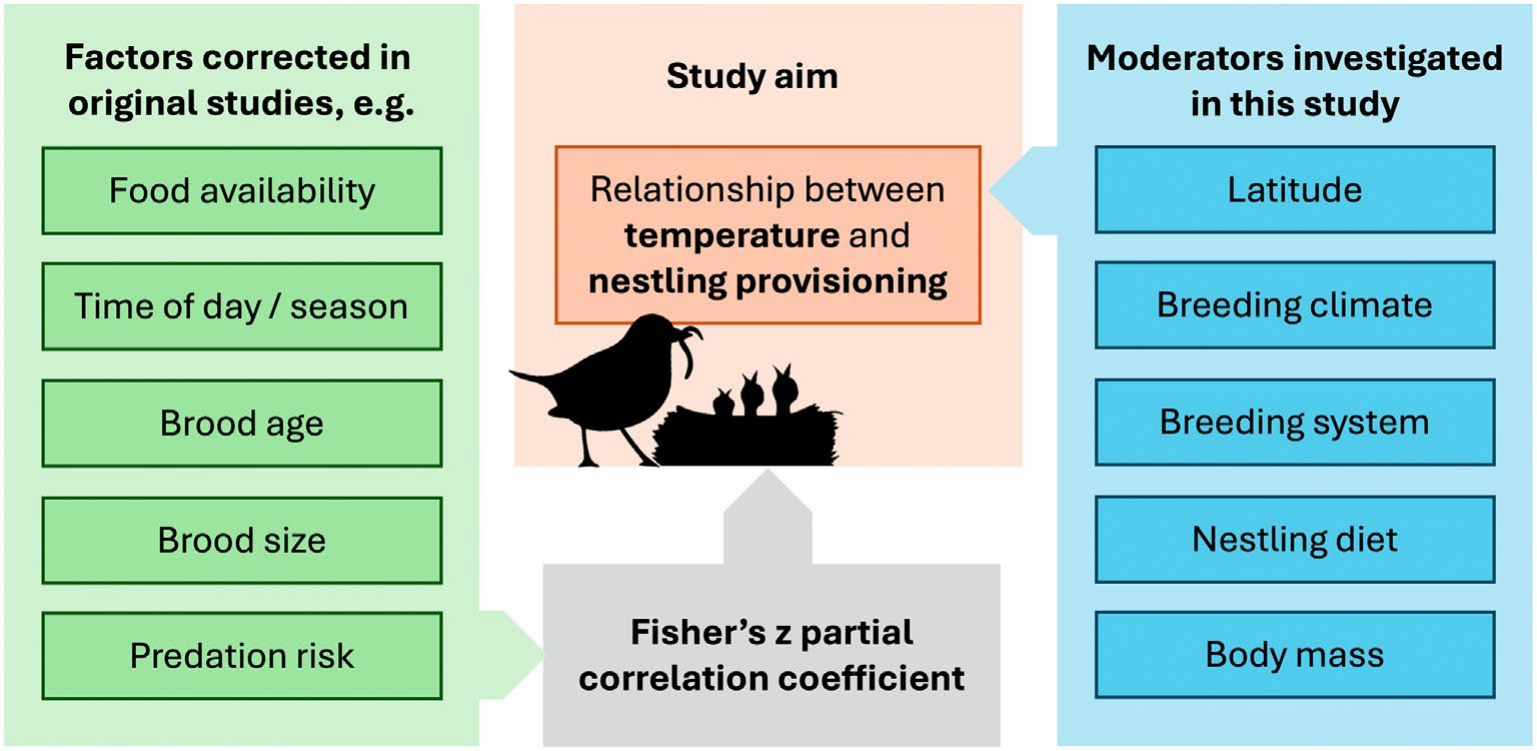
Schematic representation of the study. Factors listed in the green box represent the examples of factors often included in studies into bird parental care and provisioning rates; factors in the blue box are the moderators tested in our meta‐analysis predicted to influence the relationship between temperature and nestling provisioning. The response is measured through the Fisher's *z* partial correlation coefficient as the effect size index (grey box), accounting for the variability already explained through original factors (green box) and leaving the effect of environmental factor temperature (orange box).

Nestling provisioning and ambient temperature are both strong determinants of reproductive success (Halupka et al. 2023). Studying these two factors in tandem can offer new insights into behavioural responses to heat during reproduction, which may be relevant in our current era of climate change. Here, we synthesise the available literature in a meta‐analysis aimed at understanding the relationship between current temperature and nestling provisioning (Figure 1). We conducted this analysis using Fisher's *z* partial correlation coefficients, which were, depending on the original studies, corrected for food availability, time of day and season, brood age, brood size and predation risk. We applied a phylogenetically‐informed analysis to determine the magnitude and direction of the relationship between temperature and provisioning, while also assessing the potential impacts of five separate moderators: latitude, breeding climate, breeding system, nestling diet and body mass. Overall, we predicted that provisioning rate would be negatively correlated with ambient temperatures (i.e., during the studied days or weeks). Furthermore, based on the potential mechanisms described above, we made the following predictions related to our five moderators. First, we predicted that provisioning rates would decrease more strongly at lower latitudes, as temperatures there are generally higher and because species at lower latitudes tend to have a slower pace of life, making individual breeding attempts less valuable. Second, and in line with the previous prediction, we predicted that as temperatures increase, birds in warmer breeding climates would decrease their provisioning rates (i.e., as temperatures exceed *T*_optimum_ and reach the high end of their typically‐narrower thermal performance curves), whereas birds in colder breeding climates might maintain or even increase their provisioning rates in response to higher temperatures (i.e., as temperatures approach *T*_optimum_ of their often‐broader performance curves; Khaliq et al. 2014). Third, since helpers in cooperatively breeding species might compensate for negative effects of adverse circumstances (Jetz and Rubenstein 2011), we predicted that provisioning rates by cooperative breeders would be less affected by temperature than species that breed in pairs. Fourth, we predicted that the response to temperature would vary with nestling diet, since different food sources differ in their sensitivity to temperature (i.e., different thermal performance curves). It is currently difficult to make specific predictions about the nature of this effect because of contradicting evidence about how different food types respond to temperature. For example, high temperature can negatively influence arthropod activity and presence, but also promote arthropod reproduction and growth (Abarca and Spahn 2021; Carter et al. 2025; Hayes et al. 2024; Lehmann et al. 2020; Welti et al. 2022). Similarly, animal prey, whether invertebrates or vertebrates, might become more active when it is warmer, but might also hide during the warmest parts of the day (Angilletta Jr et al. 2002; Welti et al. 2022). In this study, we therefore do not form a priori predictions about the direction of the effect of diet on the relationship between provisioning rate and temperature, but explore different hypotheses to explain our findings. Fifth, we predicted that heavier bird species would cope less well with higher temperatures and thus reduce their parental provisioning more strongly. By evaluating these predictions, we aimed to gain a better understanding of the impact of temperature on nestling provisioning rates and identify important traits that can inform predictive models about future scenarios under ongoing global warming.

## 2 Methods

### 2.1 Compilation of Dataset

**FIGURE 2:**
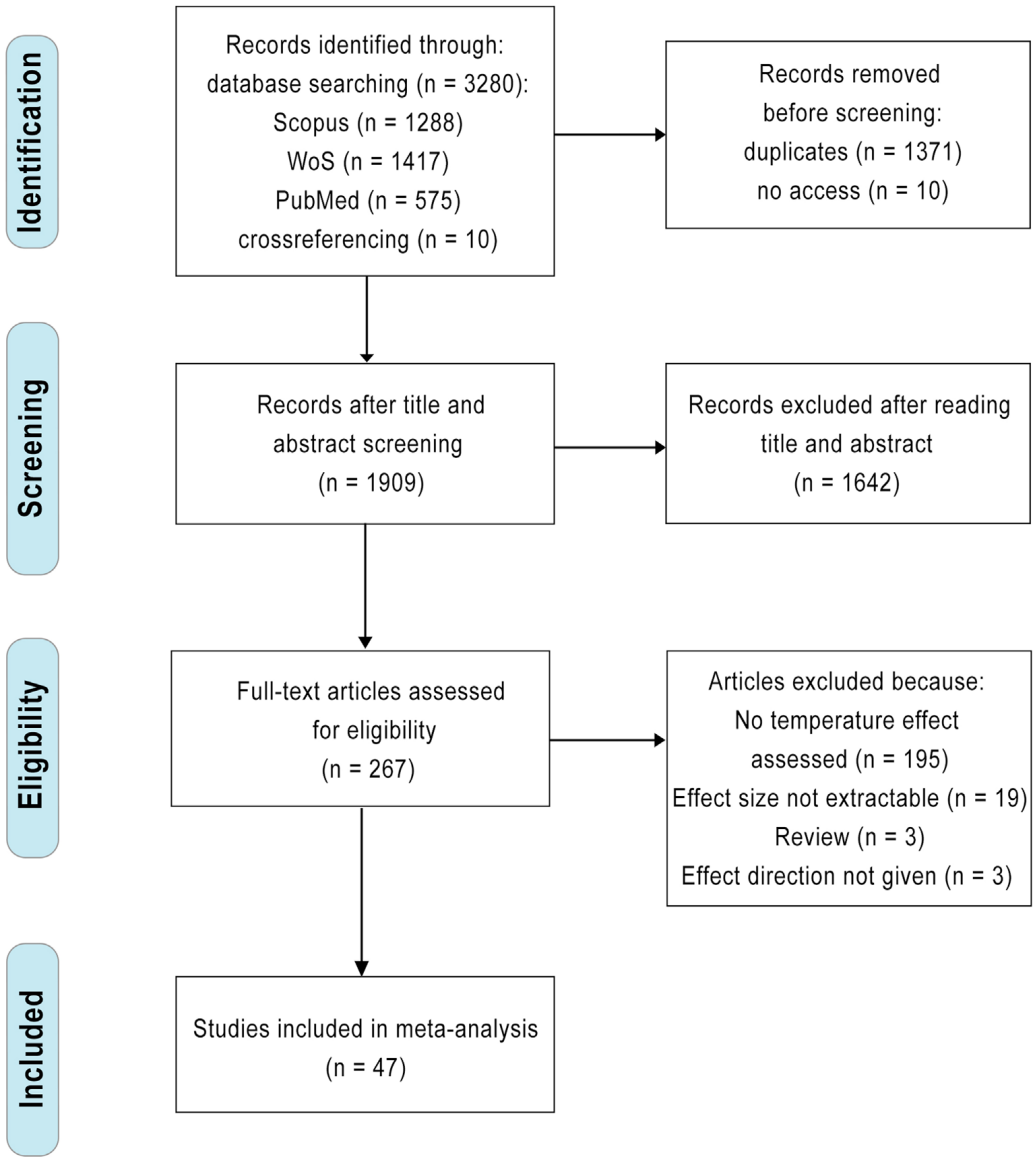
PRISMA scheme for paper selection process.

We extracted data on the relationship between ‘current’ temperature (i.e., on a daily or weekly scale depending on the study) and nestling provisioning rates of altricial bird species from peer‐reviewed publications. We obtained the publications (until December 2025) from systematic searches of three online databases: Scopus, Web of Science and PubMed. Our search query was built up following a PICO (Population, Interest, Context and Outcome) (Higgins and Green 2008) combination of search terms: (“Chick*” OR “Nestling*” OR “Hatchling*” OR “Offspring*”) AND (“*Parental care*” OR “Provision*” OR “*Chick rearing*” OR “Feeding behav*” OR “*Offspring care*”) AND (“Temperature*” OR “Climate*” OR “Weather*”). We followed the Preferred Reporting Items for Systematic reviews and Meta‐Analyses (PRISMA) for the screening and appraisal of all found records (O'Dea et al. 2021; Page et al. 2021) (Figure 2). In addition, we carefully screened the reference lists of the eligible records to ensure any additional suitable records were identified before proceeding. After removing 1371 duplicates, we screened 1909 unique records for their eligibility by assessing their title and abstract. Most (1642) publications did not fulfil our inclusion criteria (see below) and were therefore not fully read. If, based on an abstract, parental care was apparently studied but it was unclear whether provisioning rates were included, we checked the corresponding publication in more detail to determine if provisioning rates were available (e.g., as a by‐product mentioned in a supplement or box). After this step and a first round of data extraction, an additional 220 publications were removed because they did not test for a relationship between temperature and nestling provisioning rate or because data formatting did not allow for data extraction and effect size conversion (see details in PRISMA scheme (Figure 2) and Supporting Information Excel 1). The data presented in several publications necessitated special considerations. One study separately reported data of provisioning rates on day five and day 11. In this case, to be conservative, we used only the day 11 results since the two results are not independent of each other. Two other studies only reported data on bivariate correlation coefficients. Since bivariate correlations are incomparable to partial correlations, we excluded these publications (Hansen et al. 2022). Overall, 47 publications, comprising 39 different species passed all inclusion criteria (see below) and were used in our analysis.

### 2.2 Inclusion Criteria

In some studies, the response to temperature was the sole focus, but temperature effects could also be assessed secondarily (i.e., as another predictor that could influence links between provisioning rates and a third factor). For a study to be included, it needed to adhere to three inclusion criteria: (1) The study must have been conducted on an altricial bird species. We did not consider precocial bird species due to their inherently different styles of parental care. (2) The study addressed the relationship between temperature (regardless of scale/unit) and nestling provisioning rates. If a study reported ‘nest visitation rates’ as a unit while only considering the nestling period and explicitly stating that those visits equated to feeding events, then those studies were included. We did not include studies that used time of day as a proxy for temperature. (3) The study reports the relationship and direction between temperature and nestling provisioning rate as regression model output or other transformable statistical output, such as *t*‐ and *p*‐values. For experimental studies included in our dataset, we made use of results from the control groups only, thereby eliminating any (potentially biasing) experimental treatment effects.

### 2.3 Partial Correlation Coefficient

The partial correlation coefficient (*r*_p_) is a commonly used effect size index in meta‐analyses aimed at quantifying and comparing linear relationships between two variables while controlling for other variables (Aloe and Thompson 2013; Van Aert 2023). Factors corrected for in the original studies (examples in the green box of Figure 1) are integrated in the formula of the partial correlation coefficient. This effect size is thus free of the variance explained by the other factors in the original model and focused only on the correlation of interest (Nakagawa and Cuthill 2007). We applied Fisher's *z*‐transformation and used Fisher's *z*‐transformed partial correlation coefficient (*r*_zp_), which has been proposed as a better alternative to the untransformed/normal partial correlation coefficient because it is independent of the sampling variance and the sampling distribution more closely follows a normal distribution (Van Aert 2023). Effect sizes were calculated using the *escalc()* function with R package *metafor* (Viechtbauer 2010). A negative partial correlation coefficient indicates a lower provisioning rate at higher temperatures, whereas a positive value indicates a higher provisioning rate at higher temperatures.

We used the following approach for effect size computation, moving from one step to the next, as the available data required. (1) When a *t*‐value was available, we directly used it to compute *r*_zp_ using the *escalc()* function from the *metafor* package (Viechtbauer 2010). (2) If a slope (*beta*) and standard error were available, we computed the *t*‐value by dividing the slope by the standard error before converting to *r*_zp_, as in step 1. (3) If the above information was not available, we used the *p*‐value directly within the *escalc()* function. If only *p* < 0.05 was reported, we conservatively assumed *p* = 0.05.

### 2.4 Additional Moderators

**FIGURE 3:**
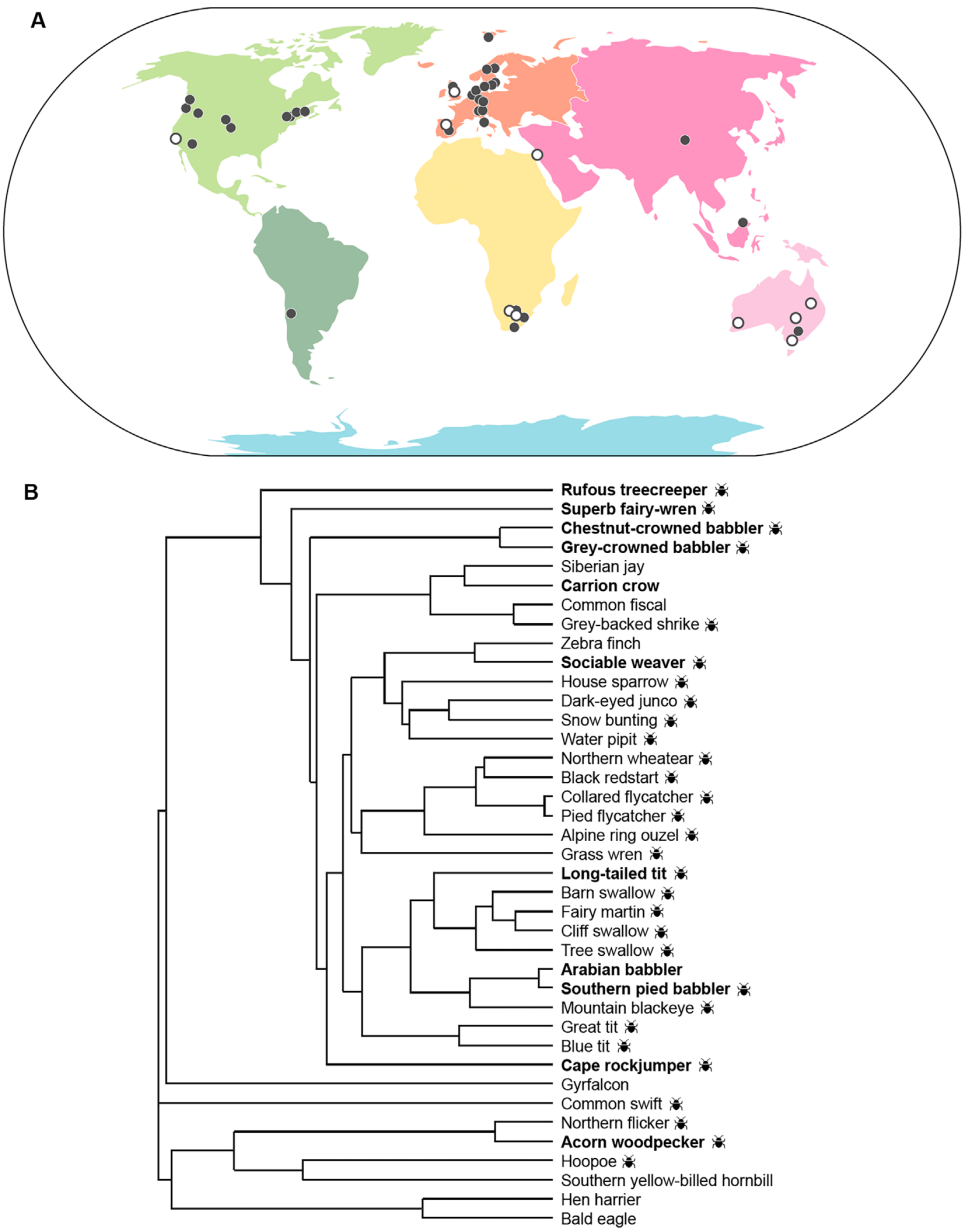
Global distribution map of studies included in our meta‐analysis. The world map (A) shows the locations of the 47 included studies. Closed points represent species that have biparental care, and open points represent cooperatively‐breeding species. The phylogenetic tree (B) shows the relationships among the included study species. Bold text indicates cooperative breeders; a symbol reflects the nestling diet as ‘exclusively insectivorous’ or no symbol, which refers to the diet as ‘not exclusively insectivorous’.

Along with the bibliographical information of each study, we recorded the common and scientific names of the species, and the sex of the provisioner(s) (male, female or both). If provisioning data were reported separately for males and females, we first calculated effect sizes for each sex and then averaged these to obtain a single value for the pair. In cases where the study reported a model or result for the pair as a whole, we used that pair‐level result directly. Details are provided in Supporting Information Excel 1. We recorded information about five moderators that might affect the link between parental care and temperature. Details of these were collected directly from each study or from separate sources as mentioned below. We recorded the geographical coordinates of the study location (latitude and longitude, expressed in decimal degrees, WGS84 ellipsoid) (Figure 3), and for the analysis used absolute latitude. Using these location details, we extracted climate information per location from ClimateCharts.net using the gridded Climatic Research Unit (CRU) Time‐series 4.07 (range 1993–2022) (Zepner et al. 2021). We calculated ‘breeding climate’ as the mean temperature across the time series for the breeding month(s) of data collection as specified in each publication (Supporting Information Excel 1). We categorized breeding system as either ‘biparental care’ or ‘cooperative breeding’, with cooperative breeding defined as breeding pairs routinely receiving help from one or more individuals in the care of offspring (Cockburn 1998). We categorized nestling diet type based on three handbooks on birds (i.e., Billerman et al. 2026; BirdLife Australia 2023; Cramp 1977; Hockey et al. 2006) and, if unavailable therein, from original sources. We classified each species as ‘exclusively insectivorous’, if parents only provisioned arthropods to nestlings, or ‘not exclusively insectivorous’ if parents supplement or exclusively provide offspring with other food items (e.g., vertebrates, seeds or fruit). This two‐level classification was chosen to maintain statistical power and prevent over‐fragmenting the data. The ‘not‐exclusively insectivorous’ group (total *n* = 9) included strict carnivores (i.e., birds of prey, *n* = 4); omnivores that feed arthropods and vertebrates (*n* = 3) or arthropods, fruit and some vertebrates (*n* = 1); and one strict granivore. Within the ‘not exclusively insectivorous’ group, two species can be viewed as dietary outliers compared to the majority that feed vertebrates plus insects: a strict granivore (zebra finch) and a fruit‐supplementing insectivore (southern yellow‐billed hornbill). We ran our analysis without these two species, and the results did not differ qualitatively. We have highlighted these two species in the corresponding figure (Figure 5) with an asterisk for clarity.

Finally, we extracted body mass of each focal species (in grams, log transformed) from Dunning (2008), or if unavailable, from Billerman et al. (2026) (i.e., Birds of the World). The references for data on characteristics of each species are provided in the Data S1.

### 2.5 Meta‐Analysis

The analyses were performed using meta‐analytical multilevel linear (mixed) effect models (Viechtbauer 2010). We chose these models to allow for the incorporation of the correlation structure due to phylogenetic relatedness among species (Cinar et al. 2022). We downloaded the phylogenetic tree (with Ericson backbone) of the 39 species in our dataset from birdtree.org (Jetz et al. 2012). We computed the branch lengths and created the correlation matrix for the phylogenetic relatedness using the *ape* package in R (Paradis et al. 2004). We fitted a random effect at the study level to capture between‐study variability and to determine whether phylogenetic relatedness improved model fit we added two more random effects within the model: one component capturing phylogenetic species‐level differences (according to the derived correlation matrix) and the other capturing non‐phylogenetic species‐level differences (i.e., pure species heterogeneity) (Model 1, Table 1) (Cinar et al. 2022). Neither of these terms significantly improved the fit of the model (AIC < 2) and therefore both were excluded. The final model therefore only has between‐study variability within the random term (Model 2, Table 1).

**TABLE 1:** Model descriptions including R code.

Model #	Description	R code
1	Base model with phylogenetic and species random effects	rma.mv(yi = zpcor, V = zpcor_var, random = list(~1 | species, ~1 | phylogeny, ~1 | study_id), *R* = list(phylogeny = correlation_mat), method = “REML”, test = “t”, dfs = “contain”, data = dat_clean)
2	Base model without phylogenetic and species random effects	rma.mv(yi = zpcor, V = zpcor_var, random = list(~1 | study_id), method = “REML”, test = “t”, dfs = “contain”, data = dat_clean, sparse = TRUE)
	Model 2 with moderator	
3 4 5 6 7	~Absolute latitude ~Breeding climate ~Breeding system ~Nestling diet ~Body mass	rma.mv(yi = zpcor, V = zpcor_var, random = list(~1 | study_id), method = “REML”, test = “t”, dfs = “contain”, data = dat_clean, sparse = TRUE, mods = ~{*Insert single moderator here}*)
	Model 2 with bias moderator	
8 9	~SE of effect sizes ~Year (mean centred)	rma.mv(yi = zpcor, V = zpcor_var, random = list(~1 | study_id), method = “REML”, test = “t”, dfs = “contain”, data = dat_clean, mods = ~{*Insert single bias moderator here}*)

To determine the overall effect of the current temperature (i.e., on a daily or weekly scale depending on the study) on nestling provisioning rates, we analysed 47 effect sizes from 47 studies, representing 39 different species. Subsequently, we assessed whether this relationship depended on latitude, breeding climate, breeding system, nestling diet and body mass by adding these moderator variables one‐by‐one to separate models (Models 3–7, Table 1). Models were fitted using restricted maximum likelihood (REML) estimation via the *rma.mv()* function from the *metafor* package in R (version 4.4.0) (Viechtbauer 2010).

We examined publication bias using an extended regression‐based model as suggested for ecological meta‐analyses with high levels of heterogeneity (Nakagawa et al. 2022). We found no indication of either a small‐study effect (i.e., where effects from studies with small effect sizes tend to be larger) or a time‐lag effect (i.e., where effect size declines with the year of publication) in our meta‐analytic dataset (Models 8–9, Table 1) (Koricheva et al. 2013; Nakagawa et al. 2022).

Finally, since we identified a single influential data point using Cook's distance via the *cooks.distance()* function from the *stats* package, we recalculated the overall estimate after exclusion of this value (Cook 1977; Fox and Weisberg 2019). Doing this did not change our overall results; thus, we present the results of analyses of the dataset including this point.

## 3 Results

### 3.1 Overall Effect of Temperature on Provisioning

**FIGURE 4:**
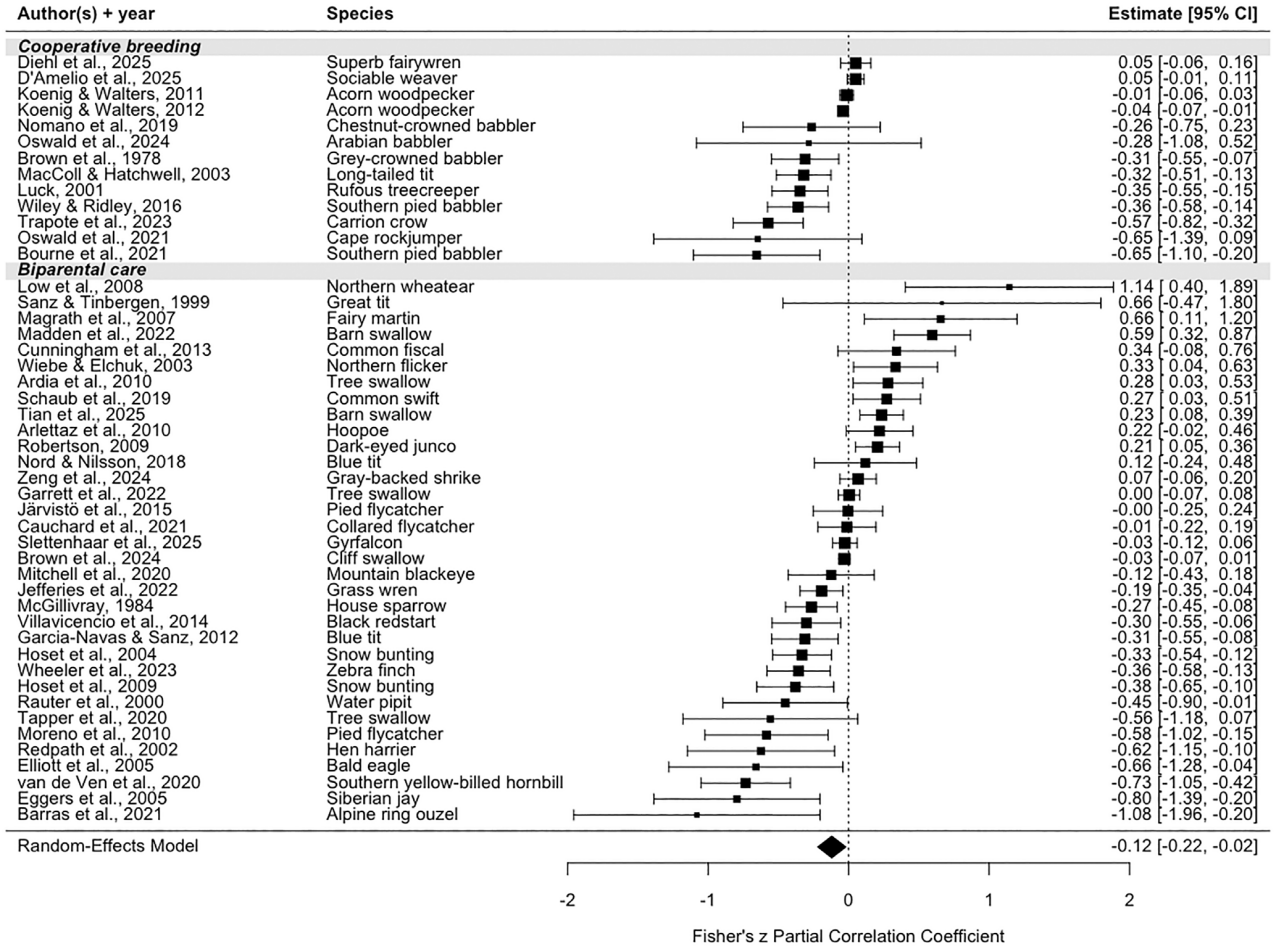
Forest plot of effect sizes of each separate study (sorted by breeding system) and the overall effect of temperature on nestling provisioning rates. These results are derived from a random‐effects model (Model 2, Table 1) including 47 studies. For each study, the Fisher's *z*‐transformed partial correlation coefficient, and 95% confidence intervals are shown. The overall, significantly negative effect is depicted by the diamond in the bottom row, indicating that across all studies and species, birds provision their nestlings less when ambient temperature increases.

Across the entire dataset, birds provisioned their nestlings significantly less at higher temperatures (*Fisher's z partial correlation coefficient*: −0.12, 95% CI UB = −0.22, LB = −0.02, *p* = 0.02, Model 2, Table 1, Figure 4). However, the effects were highly variable among studies and species. The largest fraction of the studies (21 of 47; 44.7%) showed significantly lower provisioning rates with increasing temperatures (Figure 4). The remaining studies reported significantly higher provisioning rates at higher temperatures (eight studies; 17.0%) or no significant relationship (18 studies; 38.3%).

### 3.2 Moderators of the Temperature‐Provisioning Relationship

**FIGURE 5:**
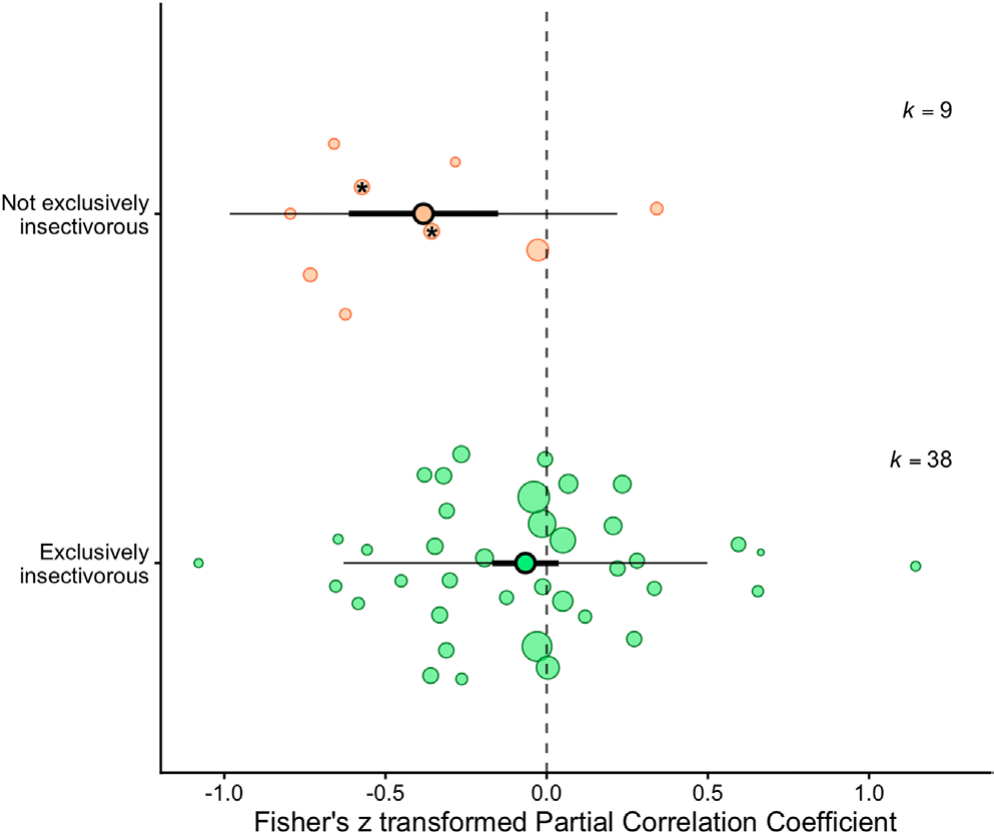
Orchard plot per nestling diet category of bird species for which the relation between nestling provisioning and ambient temperature was investigated. The coloured dots show the 47 estimates from the included studies, with ‘*k*’ being the number of effect sizes. The solid dots represent the mean estimate, the thick lines show the 95% confidence interval, and the thin lines depict the prediction interval. When bold lines do not overlap with zero the result is considered significant. The size of the circles reflects the precision (i.e., inversely proportional to the variance of the effect size), where larger bubbles represent more precise estimates (either due to larger sample sizes or lower variance). Asterisks depict two species with ‘outlier’ diets, one being granivorous and the other fruit supplemented.

**FIGURE 6:**
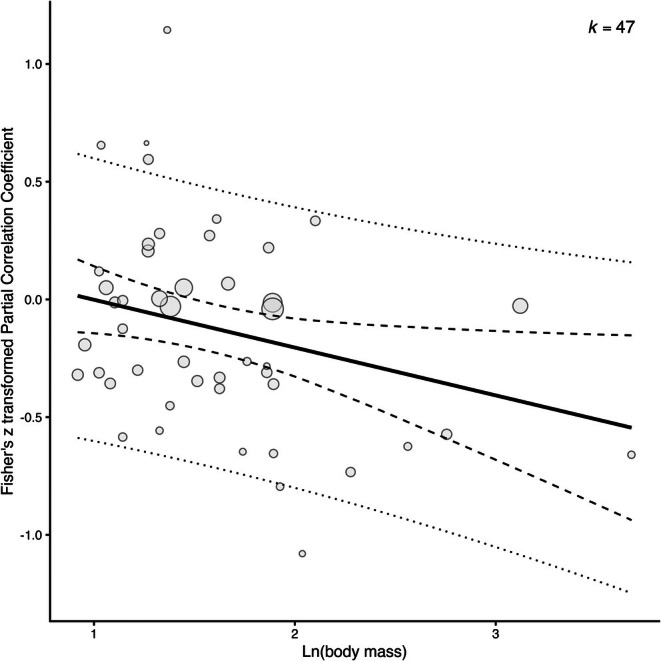
Inverse relationship between the natural logarithm of body mass and effect size estimate of 47 studies investigating the relationship between nestling provisioning and ambient temperature. The solid line represents the mean estimate, the dashed lines show the 95% confidence interval, the dotted lines display the prediction interval, and the size of the circles reflects the precision (i.e., inversely proportional to the variance of the effect size), where larger bubbles represent more precise estimates (either due to larger sample sizes or lower variance). ‘*k*’ represents the number of effect sizes.

The subsequent analyses investigating moderators that were predicted to influence the relationship between provisioning rate and temperature (Models 3–7, Table 1) gave varied results (Table 2). The relationship between temperature and provisioning rate did not vary with either latitude or breeding climate (both *p* > 0.55, Table 2). Among species with biparental care, the effect of temperature was highly variable and there was no overall correlation (*Fisher's z partial correlation coefficient*: −0.07, 95% CI UB = −0.19, LB = 0.05, *p* = 0.24, Figure 4, Table 2). However, the 13 effect sizes from 12 cooperatively breeding species showed overall lower provisioning with higher temperatures (*Fisher's z partial correlation coefficient*: −0.25, 95% CI UB = −0.43, LB = −0.06, *p* = 0.001, Figure 4, Table 2), though this relationship was significant in seven of the 13 individual studies. Of the two categories of nestling diet, only not exclusively insectivorous species showed a significant negative relationship between provisioning and temperature (*Fisher's z partial correlation coefficient*: −0.38, 95% CI UB = −0.61, LB = −0.15, *p* = 0.002, Figure 5, Table 2). Note, a qualitatively similar result was found after removing the two species that were the dietary outliers in the not exclusively insectivorous category (*p* = 0.02, with these two species excluded, asterisk in Figure 5). Among the exclusively insectivorous species the effect of temperature on provisioning strongly varied, and there was no significant overall correlation (*Fisher's z partial correlation coefficient*: −0.07, 95% CI UB = −0.17, LB = 0.04, *p* = 0.20, Figure 5, Table 2). Finally, body mass correlated negatively with the study‐specific estimates of the relationship between provisioning and temperature (*Fisher's z partial correlation coefficient*: −0.20, 95% CI UB = −0.38, LB = −0.02, *p* = 0.03, Figure 6, Table 2). Species below 200 g showed mixed responses (positive, neutral, negative) in provisioning to higher temperatures, but the four species heavier than 200 g all showed lower provisioning with higher temperatures.

**TABLE 2:** Results of five meta‐analytic single moderator models.

Moderator	Category	Estimate	95% CI	*p*
~Latitude		0.0007	−0.007 to 0.008	0.84
~Breeding climate		0.005	−0.01 to 0.02	0.55
~Breeding system	Biparental care	−0.07	−0.19 to 0.05	0.24
	**Cooperative breeding**	**−0.25**	**−0.43 to −0.06**	**0.001**
~Nestling diet	**Not exclusively insectivorous**	**−0.38**	**−0.61 to −0.15**	**0.002**
	Exclusively insectivorous	−0.07	−0.17 to 0.04	0.20
~Body mass		**−0.20**	**−0.38 to −0.02**	**0.03**

*Note:* Models examining how environmental factors and species traits influence the effect of temperature on nestling provisioning rates (response: Fisher's *z*‐transformed correlation coefficients, zpcor). Models were fitted using random‐effects meta‐analysis (rma.mv(), metafor package (Viechtbauer 2010), with study identity included as a random term). The analyses are based on 47 effect sizes from 39 species. Significant effects (*p* < 0.05) are highlighted in bold.

## 4 Discussion

Overall, our meta‐analysis reveals that birds across the globe decrease their nestling provisioning rates in response to increases in current temperature. Yet, while this overall effect is significant, not all species appear to be affected similarly. To different extents, ecological and species‐specific characteristics affect how parental provisioning changes with increases in current temperature.

### 4.1 Latitude and Breeding Climate

Latitude and breeding climate each explained very little of the variation in the relationship between temperature and nestling provisioning rate, in contrast to our predictions. On the level of individual studies, several bird species have been shown to feed their nestlings less when temperatures increase. This is particularly the case in regions with generally high, and strongly fluctuating temperatures, such as in Australia and Southern Africa (Wheeler et al. 2025; Wiley and Ridley 2016). However, cold specialists, such as snow buntings ( *Plectrophenax nivalis* ) in the very northern parts of Norway (Hoset et al. 2004, 2009), have also been shown to decrease their provisioning rates in response to temperature. Snow buntings can be at risk of overheating while in flight at ambient temperatures as low as 9°C (O'Connor et al. 2024). In some species in temperate regions, the opposite relationship (i.e., higher provisioning rates with higher temperatures) has been observed (Cauchard et al. 2021; Low et al. 2008; Nord and Nilsson 2019). Such a pattern might arise by parents being released from the brooding needs of their young while still being far from their own thermal‐tolerance maxima, or, alternatively, simply from increases in food at higher temperature. Alternatively, this may reflect that observations were conducted at temperatures below species‐specific thermal optima (*T*_optimum_), where performance is not yet constrained, rather than indicating an absence of temperature effects per se. It is important to note that our interpretation of latitudinal effects on the relation between temperature and parental provisioning need to be made with care, since species from the equatorial regions were not well represented in our dataset. This highlights the need for more empirical data from lower latitudes and warmer regions. In any case, the variation across species does not appear to be explained by differences in latitude or breeding climate.

### 4.2 Breeding System

Species with biparental care varyingly responded to increased daily temperatures by increasing, decreasing or maintaining parental provisioning rates. While species with biparental care showed a large range of different responses between parental provisioning and temperature, cooperative breeders responded, overall across species, to increased temperatures by on average reducing parental provisioning, even though only about half of them did so significantly at the level of individual study (see Figure 4). This overall significant negative response contrasted with our prediction that cooperative breeders would be less affected by temperature. One possibly relevant difference between species that are cooperative breeders and those exhibiting biparental care is the level of diversity within these two groups. As a group, cooperative breeders generally tend to be long lived (Covas and Griesser 2007), have distribution ranges at lower latitudes, and share other ecological characteristics (Jetz and Rubenstein 2011). Species with biparental care are much more varied. In the dataset analysed here, species ranged in body size (from 12 g zebra finches (*Taeniopygia castanotis*) to ca. 5 kg bald eagles ( *Haliaeetus leucocephalus* )), covered a wide range of ecological niches (from montane to urban), varied in their specialisation (from highly specialised to very generalist), and exhibited diverse life‐history characteristics (from slow to fast pace‐of‐life). For example, as part of life‐history characteristics, longevity influences how many times within a lifetime a brood can be produced, affecting the value of a given brood (Soriano‐Redondo et al. 2020; Wiersma et al. 2007). In short‐lived species the uncertainty of future breeding opportunities increases the value of current reproduction (Ghalambor and Martin 2001). In contrast, long‐lived species are expected to value future reproduction more and thus have evolved to make different trade‐offs between current and future investment (Dobson and Jouventin 2010; Griesser et al. 2017). In this latter case, selection is likely stronger for the parents to maintain in good condition across multiple breeding events; hence, when faced with a challenge like hotter than normal weather, these parents may be more prone to reducing nestling provisioning rates.

Across the 13 studies on cooperative breeders, nestling provisioning rates overall (though not always significantly) decreased with temperature. Cooperative breeders often inhabit, and are thus adapted to, areas with harsh or variable conditions (Rubenstein and Lovette 2007; Jetz and Rubenstein 2011) and having more individuals contribute to offspring care may enable such species to better cope with heat and other environmental challenges (Komdeur and Ma 2021). Reduced provisioning by cooperative breeders at higher temperatures may reflect several non‐exclusive mechanisms. First, cooperative breeders may be able to afford temporary reductions during the hottest periods because more individuals can better compensate for this when they resume provisioning when conditions become more suitable (e.g., earlier or later during the day or during cooler periods). Second, relatively young and inexperienced helpers, may be less able or willing to maintain provisioning when circumstances get more difficult. Indeed, Australian chestnut‐crowned babbler ( *Pomatostomus ruficeps* ) helpers reduced their visitation rates on hot days, and more helpers did not translate to a higher overall provisioning rate (Nomano et al. 2019). Third, in the generally warm and harsh environments inhabited by cooperative breeders, birds may be operating near or above their thermal optima (*T*_optimum_), resulting in reduced provisioning rates despite potential fitness costs to offspring. Consistent with this, species occurring in hotter environments may be more frequently exposed to temperatures approaching or exceeding their thermal optima, which could contribute to the stronger negative responses observed; however, comparable estimates of thermal limits across all species are currently lacking. Finally, cooperative breeders may re‐nest more quickly after failure (Ridley and van den Heuvel 2012), meaning that the loss of a brood due to extreme temperatures may have smaller negative impacts on fitness than in biparental species. Together, these patterns suggest that reduced provisioning at high temperatures may be a flexible response, rather than a sign of limited coping ability, especially if group members can later compensate for reduced provisioning.

### 4.3 Nestling Diet

Among the exclusively insectivorous bird species, the effect of temperature on provisioning strongly varied, and there was no significant overall correlation. While extreme temperatures can lead to a reduction in prey activity and presence (Carter et al. 2025; Hayes et al. 2024; Welti et al. 2022), higher temperatures may also promote arthropod reproduction and growth, leading to prey that is more abundant, larger or both (Abarca and Spahn 2021; Lehmann et al. 2020). Regardless of such specific potentially contrasting effects, weather conditions, including temperature, can have strong cascading effects through prey availability. Annual breeding success of a population of European bee‐eaters ( *Merops apiaster* ) in Germany was 32% higher following a dry and hot summer, mainly because of the increased availability of large flying insects (Arbeiter et al. 2016). Subtle diet alterations or the provisioning of larger prey items may be a way for species to allow a reduction in provisioning rates while at the same time meeting the nutritional demands of their nestlings. Arthropods vary largely in their nutritional value, and higher temperatures may allow greater selection of the nutritional quality of prey (Razeng and Watson 2015). Depending on the dietary requirements of species and their nestlings, there might be strategies that allow birds to change their nestling provisioning rate as well as the type of prey provisioned. For generalist insectivorous species, this might mean they compromise on the quantity of food provided but not on the quality (which could even increase). The finding that insectivorous species did not show any systematic response in their provisioning rate during hotter periods is likely the result of multiple interacting factors, which require further study to disentangle.

The not exclusively insectivorous species showed a significant provisioning rate response to temperature, in this case negative. This result is somewhat surprising, and furthermore hard to interpret given the high diversity of dietary types represented within this group. Given the small sample size and high diversity, limited conclusions can be drawn. However, the fact that we found no overall effect of temperature on provisioning rate in exclusive insectivores, combined with the fact that excluding the two dietary outliers named above from the not exclusively insectivorous group did not change the result, we can tentatively suggest that the significant effect of temperature in this group is driven by species feeding large live prey to their nestlings. Perhaps this negative relationship might be a consequence of the energetic demands associated with capturing typically larger vertebrate prey or their accessibility. Ectothermic reptiles are typically more active during warm temperatures (Angilletta Jr et al. 2002), but this might make them more difficult to catch due to their increased speed of movement. Endothermic mammals, which are highly sensitive to overheating (McKechnie and Wolf 2019), typically take shelter in the hottest parts of the day. The predicted effect of temperature on these two types of prey species might explain why bird species feeding their offspring non‐insectivorous diets respond more strongly to temperature, but we encourage additional studies, especially in non‐insectivore species, to explicitly investigate the effect of food availability on the effect that temperature has on the food provisioning of offspring.

### 4.4 Body Mass

For species with higher body mass (i.e., bigger and heavier birds), the effect of temperature on provisioning rate became more negative. Thus, compared to lighter species, heavier species may be more susceptible not only to bouts of hot weather, but also a warming climate. The observed correlation with body mass could be explained by several different mechanisms, which require further study. First, larger altricial birds may rely on types and quantities of food that are more energetically demanding to acquire, such as discussed in Section 4.3. Second, basic physiological scaling laws may underlie this result: the smaller surface‐to‐volume ratio of larger species obstructs heat loss (James 1970; Olson et al. 2009). Our results suggest that the amount of parental care through nestling provisioning may be under strong pressure once temperatures rise, particularly for larger species.

### 4.5 General Conclusions

We have identified factors, including breeding system, diet and body mass, that are associated with temperature‐related changes in nestling provisioning rates and thus that can be useful when assessing a given species' vulnerability to climate change. The high degree of variation in how these traits modify the relationship between provisioning and temperature among bird species, however, suggests that additional factors, or complex interactions among our separately analysed factors, underlie how breeding pairs or groups change provisioning behaviour when confronted with higher temperatures. While the number of relevant studies limited our current analyses, assessing, for example, the role of pace of life and longevity could be a promising avenue for future work as more studies become available. At the same time, among individuals of the same species, the availability of thermal refugia or microclimates can result in very different responses to hot weather if the heat has differential impacts on birds' nests, territories or home ranges (Cunningham et al. 2021; Kim et al. 2022; Suggitt et al. 2018). Furthermore, birds may differ in their motivations and abilities (i.e., different strategies related to food quality, different thermal tolerances and parental investment choices) to buffer the impacts of environmental challenges on themselves and their offspring. Because, for example, larger, more nutrient‐dense or more hydrating food might be prioritised but provided to nestlings less often, an increased focus on what is being delivered to nestlings is warranted in future studies. More detailed within‐species studies of nestling provisioning, and parental care more generally, are needed to reveal if and how birds can cope with changing environmental conditions. Potentially less‐conspicuous adaptations may greatly impact parental strategies under challenging conditions and the distribution of costs between parents and their offspring. Ultimately, such insights are essential for well‐founded and comprehensive models aimed at understanding and predicting the effects of climate change on global bird populations.

## Author Contributions

**Kat Bebbington:** supervision, conceptualization, writing – review and editing. **Kevin D. Matson:** supervision, conceptualization, writing – review and editing, visualization. **Elke Molenaar:** conceptualization, investigation, writing – original draft, methodology, visualization, formal analysis. **Sjouke A. Kingma:** validation, supervision, conceptualization, writing – review and editing, project administration, visualization, methodology.

## Funding

This work was supported by Wageningen Institute of Animal Sciences, Wageningen University and Research.

## Conflicts of Interest

The authors declare no conflicts of interest.

## Supporting information


**Data S1:** gcb70871‐sup‐0001‐DataS1.zip.

## Data Availability

The complete dataset, paper selection file and script for analyses are all stored and openly available through Zenodo. DOI: http://doi.org/10.5281/zenodo.19451919.
